# Everyday Activities for Children with Mitochondrial Disorder: A Retrospective Chart Review

**DOI:** 10.1155/2018/5716947

**Published:** 2018-06-06

**Authors:** Marieke Lindenschot, Imelda J. M. de Groot, Saskia Koene, Ton Satink, Esther M. J. Steultjens, Maria W. G. Nijhuis-van der Sanden

**Affiliations:** ^1^Department IQ Healthcare, Research Institute for Health Sciences, Radboud University Medical Center Nijmegen, Postbus 9101, 6500 HB Nijmegen, Netherlands; ^2^Department of Occupational Therapy, HAN University of Applied Sciences, Postbus 6960, 6503 GL Nijmegen, Netherlands; ^3^Research Group Neurorehabilitation, HAN University of Applied Sciences, Nijmegen, Netherlands; ^4^Department of Rehabilitation, Donders Center for Neuroscience, Radboud University Medical Center Nijmegen, Postbus 9101, 6500 HB Nijmegen, Netherlands; ^5^Department of Pediatrics, Radboud Center for Mitochondrial Medicine, Radboud University Medical Center Nijmegen, Geert Grooteplein Zuid 10, 6525 GA Nijmegen, Netherlands; ^6^European Masters of Science in Occupational Therapy, Amsterdam University of Applied Sciences, Amsterdam, Netherlands

## Abstract

**Background:**

Engagement in everyday activities is important for the health and wellbeing of children. Children with mitochondrial disorders have impaired energy production leading to limitations in activity. It is unknown which activities these children perform and if the nature of activities of low-functioning children differs from average-functioning children. Therefore, this pilot study explored the activities reported in patient records of a heterogeneous group of children with genetically confirmed mitochondrial disorders.

**Methods:**

A retrospective qualitative directed content analysis by health care professionals reported activities (as part of their professional reasoning obligations) in hospital patient records of children with mitochondrial disorder.

**Results:**

Seventeen patient records, presenting notes on capacities and performed activities, showed an overview of everyday activities that covered the categories: self-care, house chores, therapy, school, computing, hobby, play, sports, and mobility/transport. The activity categories of low-functioning children did not differ from average-functioning children, although descriptions of specific activities differed between groups.

**Conclusion:**

This pilot exploration indicates that the types of activities that children with mitochondrial disorders perform are not necessarily linked to the child's impairments. However, differences in levels of independence, assistive device usage, and energy costs seem to exist. Future research should address the child's perspective on, and meaning of, activity performances.

## 1. Introduction

Performing everyday activities is viewed as an important factor for the wellbeing of an individual and is an important determinant of health [1–3]. If children are unable to be engaged in everyday activities, they do not have the opportunity to explore their environment and are less able to grow up as unique individuals [4]. Although children with disabilities might enjoy the same activities and have similar desires as their healthy developing peers [5, 6], they can experience limitations in activities, leading to restrictions in their participation in daily life.

Mitochondrial disorders, with an estimated prevalence of 23 in 100,000, are one of the most common inherited errors of metabolism [7]. Mitochondrial disorders can be caused by mutation of genes encoded by either nuclear DNA (nDNA) or mitochondrial DNA (mtDNA) [8]. Mutations have been identified in more than 230 different genes [8, 9], and the number of identified genes associated with mitochondrial disorder is continuously increasing [10]. This genetic heterogeneity is also reflected in the clinical phenotypes associated with mitochondrial disorder, with a large range of symptoms and impairments [11–13]. It was previously found that children (when we talk about children in this article, we mean children and adolescents in the age of 0–18 years) with mitochondrial disorders are less physically active and engage less in vigorous activities compared to healthy peers [14]. Children with a mitochondrial disorder experience these limitations in performing everyday activities due to motor and cognitive impairments including muscle weakness, balance problems, concentration problems, and intellectual disability [13]. These impairments have consequences for daily living activities and participation. The extent of perceived limitations in activities and participation of children with mitochondrial disorders varies from none to extremely high [13]. While some children remain in a mainstream school and achieve normal milestones, others barely interact with their environment [15].

Although literature states that children with mitochondrial disorder experience limitations in activities, a clear overview of their everyday activities is lacking. It is commonly expected that the nature of activities differs based on the global functioning of the child. Knowledge of these possible differences and similarities is important to choose or develop assessments for this population, to evaluate the outcome of treatment on participation, and to be able to tailor the assessments and treatment to the child's and parent's specific wishes. As healthcare professionals make clinical decisions based on information about the child's capacities and activities, we expect that information concerning the activities of the child is reported in patient records. Therefore, this pilot study is aimed at exploring which everyday activities of children with genetically confirmed mitochondrial disorders are reported in patient records by health care professionals and understanding the profile of activities these children participate in.

## 2. Methods

A retrospective chart review [16, 17] was performed. A directed content analysis [18] was used to explore which everyday activities were reported in patient records and to explore if they differed among the functional profiles of the children.

### 2.1. Participants

The participants of this study were children (0–18 years) with a genetically confirmed mitochondrial disorder who were periodically evaluated in the so-called “MitoRoute,” a multidisciplinary, medical, and allied health care screening program at Radboud Center for Mitochondrial Medicine (RCMM), Radboud University Medical Center (Radboudumc), Nijmegen. During MitoRoute, the children were evaluated by a team consisting of several physicians of different disciplines and allied health professionals, including a physical therapist, occupational therapist, speech therapist, dietitian, social worker, and psychologist. The goal of this screening program was to establish a detailed overview of the consequences of the disorder for the child on different dimensions of the International Classification of Functioning, Disability and Health for Children and Youth (ICF-CY) [19], to prevent and detect possible complications and formulate treatment advice. The patient records of all children with a genetically proven mitochondrial disorder who were screened from May 2015 to May 2016 in the Radboudumc were included in this study.

### 2.2. Data Collection

Qualitative data concerning activities of children with mitochondrial disorders were extracted from clinical notes of (allied) health care professionals (MitoRoute) in the electronic patient records. The patient records contained unprompted written information relevant to the assessment and conversations performed by (allied) health care professionals, as they were not asked to collect data specific to this study.

### 2.3. Stratification

As motor function, cognitive abilities, and communication influence participation in activities [20, 21], these characteristics were used to construct functional capacity profiles of the children. To ensure the quality of the stratification into the profiles, a log was kept documenting thought processes and tracked decision-making. Member checking with health care professionals was used to verify the profiles. Functional capacities were stratified as the following: *Cognitive development* (based on the age-related norm-referenced test used for each child): 1 = extremely low cognitive functioning, 2 = low cognitive functioning, 3 = average cognitive functioning, and 4 = good cognitive functioning; *Ambulation* (Hoffer scale [22], with the normal ambulation level added by Schoenmakers et al. [23]): 1 = nonambulation, 2 = nonfunctional ambulation, 3 = household ambulation, 4 = community ambulation, and 5 = normal ambulation; and *Speech abilities* (Radboud dysarthria assessment [24]): 1 = no oral communication possible, 2 = communication with a known person, 3 = frequent repetitions (frequently repeating themselves to guarantee that they will be understood), 4 = incidental repetitions, 5 = effective despite small problems, and 6 = effective communication.

Other patient characteristics collected included gender, age, gross motor development (based on norm-referenced tests), performance in daily activities (based on Pediatric Evaluation of Disabilities Inventory (PEDI) [25]), and fatigue (measured with Pediatric Quality of Life Inventory Multidimensional Fatigue Scale (PedsQL-MFS) [26]). These characteristics were not used in defining the profiles but were used as descriptive outcomes linked to each profile.

### 2.4. Profiles Based on Functional Capacities

Three profiles could be defined based on the functional capacities of the children:

*Global low functioning*: children with an extremely low or low cognitive developmental level and limited motor functioning which lead to nonfunctional outdoor ambulation and nonfunctional communication.

*Low cognitive functioning with a moderate to normal ambulation*: children with extremely low or low cognitive developmental level and some limitations in motor functioning but able to have functional ambulation. Speech abilities in this profile differ among the children.

*Global moderate functioning*: children with average cognitive developmental level and some limitations in motor functioning but able to have functional ambulation. Speech abilities are sufficient to make themselves understandable.

### 2.5. Data Analysis

In this exploratory study, the method of directed content analysis [18] was used on the text fragments extracted from the patient records [27–29]. The text fragments were focused on information reported by the health care professionals regarding the experiences of children and parents in their daily life situations. Before starting the analysis of the patient records, two preliminary steps were conducted:

1. Identify the key concept “activity” as initial coding category
2. Operationalize the concept “activity” using theory

In step 2 of the data analysis, the term “activity” was defined based on the ICF-CY [19] and occupational therapy literature [30–32] as an activity that is the meaningful targeted performance of tasks and/or actions by an individual, like playing guitar, dressing yourself, eating, and cycling.

For the analysis of the patient records, the following steps were conducted by one of the researchers:

1. Read text and highlight all text that appears to represent activity
2. Code the highlighted text with codes fitting the concept activity
3. Categorize the codes into groups of activities
4. Organize the categories (the different groups of activities) into three occupational performance areas of Reed and Sanderson [33]: personal maintenance, productivity, and leisure

The procedure of the analysis and the result of steps 4 and 6 were peer reviewed by the fourth author (TS).

### 2.6. Ethical Considerations

The data used in this research were obtained during the medical care for which the child and parents gave approval to use anonymously for research (ethical board approval number 2013-287). Data were deidentified before analysis. The research was conducted conforming to the ethical principles of the Declaration of Helsinki [34].

## 3. Results

Patient records of seventeen children (five girls and twelve boys) were included. The age of the children varied from 4 to 18 years old. Eight children had a mutation on their nuclear DNA and nine children on mitochondrial DNA. Five children demonstrated a global low-functioning profile, five children demonstrated a low cognitive-functioning profile with a moderate to normal ambulation, and seven children demonstrated a global moderate-functioning profile. The clinical and genetic characteristics of the children are presented in Table 1. More clinically relevant priorities hindered scheduling of the fatigue assessment in two cases. Therefore, they were not available in the chart.

**Figure 1:**
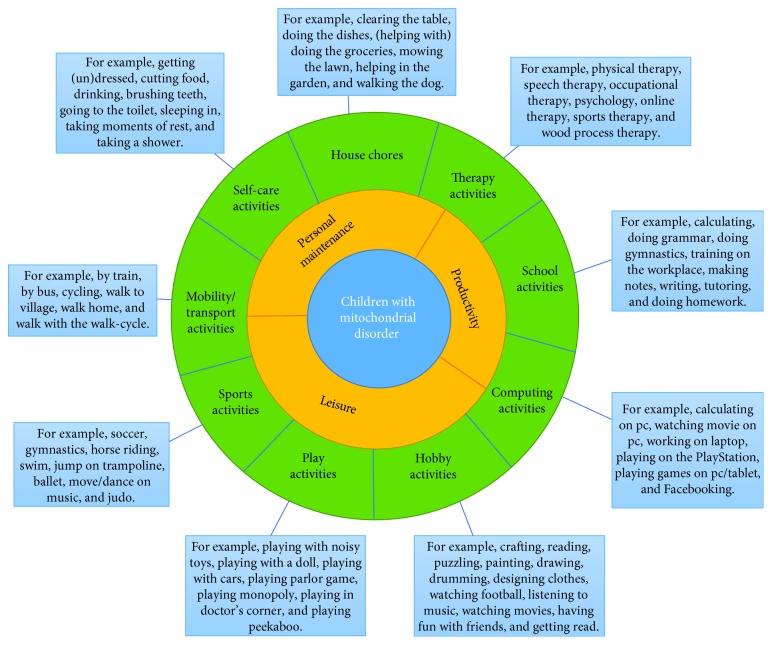
Activities, activity categories, and occupational performance areas of children with mitochondrial disorder.

As mentioned before, the activities were organized in three occupational performance areas of Reed and Sanderson: personal maintenance, productivity, and leisure [33]. Personal maintenance activities permit a person to maintain individual life support needs, for example, getting dressed or taking a shower. Productivity activities assist society to facilitate each person to meet individual needs by the use of collective resources, for example, going to work or doing homework. Leisure activities permit the individual to express needs for creative outlet, for example, drawing or swimming [33]. The outcome of step 5 of the analysis was the activity categories: self-care, house chores, therapy, school, computing, hobby, play, sports, and mobility/transport. Some activity categories could be placed under two occupational performance areas as the meaning or value of the activity was not noted. For instance, mobility/transport activities could be performed as personal maintenance but also as leisure. The results, including any double placement, are presented in Figure 1.

### 3.1. Personal Maintenance

The area under personal maintenance included all the activities that children do to take care of themselves and organize their lives. It comprises the following categories: self-care, house chores, and mobility/transport. Across the three profiles, the (allied) health care professionals reported on all three different categories in personal maintenance. The patient records showed similarities across the profiles in self-care activities like showering, eating, taking rest, and getting dressed. In the category mobility/transport activities, cycling and taking the bus were commonly reported activities. Although in this category and the category house chores, the activities showed more differences among the profiles. The activities varied from doing the dishes to mowing the lawn.

The notes in the patient records indicate that there was a difference in the support or assistance that the children received when they performed the activity. For instance, the activity eating was mentioned as “*she can eat independently*,” “*with spreading butter and cutting he gets help*,” and “*he uses an eating device which makes him able to move the cutlery independently to his mouth*.” In several activities, receiving assistance or using assistive devices or adaptations were mentioned. For instance, getting dressed and showering were mentioned as “with help” or as “independent.” Occasionally, no notes about the support or assistance were written.

### 3.2. Productivity

The area under productivity featured all the activities that children do to learn and work. It included the following categories: school activities and therapy activities. However, therapy activities can also be seen as personal maintenance. Across the three profiles the (allied) health care professionals documented notes that fit within both categories in productivity. Children within the global low-functioning profile all attended to special education, some with training for future work positions: “*He goes to secondary special education, while doing two days of workplace training in an employment center for people with intellectual disabilities*.” Three children from the low cognitive level with a moderate to normal ambulation profile attended to special education, while two children attended to regular education. Two of the children with the global moderate-functioning profile attended to special education, while five attended to regular education. Sometimes, small adaptations were made: “*She goes to regular education, but when she is too tired she stays at home for the afternoon*.” This quote also illustrate that the energy level is used as a deciding factor in performing or avoiding the activity. Notes about the energy level as the deciding factor were present in all occupational performance areas. Although the type of education varied from special to regular education, the range of activities did not differ among the three profiles. Most children attended classes that included writing, spelling, and calculating, as well as physical and/or occupational therapy.

### 3.3. Leisure

The area under leisure featured all the activities that children do to play or occupy their free time. It included the following categories: computing, hobbies, play, and sports. In each of the three profiles, the (allied) health care professionals noted all four different categories in leisure. The physically passive activities were reported more in the global low-functioning profile than the other two profiles. For instance, it was mentioned that: “*After school he is tired and uses his laptop or tablet to play games, uses Facebook or watches movies*.” In the profiles of children with a better ambulation, there were more regular physically active activities, for instance, “*After finishing his homework he usually goes outside and jumps on the trampoline or plays soccer*.” The analysis showed that the children performed individual leisure activities, for example, horse riding, jumping on the trampoline, watching football, or going to Bible study. Some activities were more popular, including swimming, gymnastics, watching television, or a movie.

## 4. Discussion

This study provides a first overview of the activity categories of children with a mitochondrial disorder. Three occupational performance areas: personal maintenance, productivity, and leisure [33] comprised, as reported by allied health professionals in patient records, nine activity categories: self-care, house chores, therapy, school, computing, hobby, play, sports, and mobility/transport. Contrary to our expectations, we found that all activity categories were present in each global-functioning profile. This is congruent with the guiding principles for children's rehabilitation in the Netherlands, which stipulate that children with disabilities are full participants in everyday life [35].

The activities performed by children with mitochondrial disorders, as identified in this study, resemble the activities of typically developing children identified in the PACS Pediatric Activity Card Sort: the PACS [36], with one exception: “therapy activities”. The PACS was developed based on typical activities of childhood that are relevant for all, not only for typically developing children. This means that this instrument has potential for use as an outcome measure for children with mitochondrial disorders. Although the current study did not compare the activities of children with mitochondrial disorders to healthy peers, the results appear to echo the findings of Shields et al. [6] and King et al. [37] who found that there were more similarities than differences in the participation of children with intellectual disabilities and their typically developing peers. The current findings and existing literature emphasize the fact that the type of activities a child engages in is not necessarily linked to the child's disabilities, but the nature of the performance differs.

Within and between the activities, we found diversity in the amount of support or assistance provided and the equipment used among the children to facilitate successful participation. For example, cycling was reported as performed differently, for example, on a regular bike, a walk-cycle or duo bicycle and with guidance or independently. Also, eating was an activity that was reported as performed independently, with help or with an eating device. It can be reasoned that the regular use of the PEDI [25] could have influenced the notes in the patient files as assistive devices or support is measured with this assessment. However, only the occupational therapist used the PEDI, the other (allied) health professionals did not, yet they also reported the amount of assistance or assistive devices. In addition to the notes on independence, there were also notes about energy levels as a deciding factor in performing or avoiding activities. This is not surprising, given that in the professional literature describing the care of children with mitochondrial disorder, fatigue is a frequently mentioned burden [13]. Although the study design did not allow for a comparison between the amount of assistance or adaptations to energy levels in performing activities, there are indications that differences exist in levels of independence, assistive devices, and energy costs across the children. When children have limited abilities or are more dependent, a higher burden is placed on parents to facilitate participation and autonomy [38, 39]. This suggests that future research should focus on the differences in levels of independence and assistive devices in relation to energy costs and caregiver burden.

We found clear activity categories that represent the different activities and could be organized into frequently used occupational performance areas [33] as described in the literature. The activity categories are also consistent with the (sub)domains of the ICF-CY [19]. We organized the activities in categories based on the typical purpose of the activities for children, which best fit the three occupational performance areas of Reed and Sanderson [33]. It was noticed that some activity categories could be linked to more occupational performance areas than others. For instance, computing activities are used to learn or work (productivity) but also for leisure. This supports the idea that the meaning of the activity can be viewed from different perspectives, can differ for each child and that activity categories are not solely linked to a certain occupational performance area [40]. To confirm to which activity categories an activity belongs, we need to gain insight in the perspectives of the children on the individual meaning each activity has for them. The categorization of activities by the children themselves is therefore suitable for use in future research.

Using patient records resulted in some limitations in interpreting the findings. As the written information was unprompted, not every (allied) health professional reported the same information. Another consequence of using patient records is that it was not always clear if the activities that were performed during therapy had a relationship to the activities that the children performed and experienced as meaningful in the daily life situation. For example, “*The child found the pencils and immediately started coloring*.” This note about a test situation in one of the patient records can imply that the child likes to color, but this is disputable. Outcomes of test situations cannot be seen as representative of the child's daily life [41]. In this research, we focused on activity performance, which describes what a person actually does in daily life, expressing the individual's involvement in a life situation [42]. Interviewing the children themselves would provide knowledge about their self-reported activity performance and experience in daily life.

Another limitation of the study is the small sample size and stratification of the sample into three global-functioning profiles. However, the sample size overall is suitable for an exploratory study taking into account the low prevalence of the disease. In each profile, there were even smaller numbers of participants; however, this was necessary to address the difference in functioning levels of the children. Prediction of the activity profile based on the level of impairments in this population is difficult given the unpredictable nature of the condition. Nonetheless, our study showed a large diversity of functional capacities, which indicates that we covered the broad spectrum of impairments and limitations seen in this population [43]. Stratification into the functional capacity profiles enhanced the insight into activity categories of the total study population and each profile separately. Conversely, a selection bias might have occurred because solely reports of children within the specific MitoRoute were included. Pediatricians refer all children with mitochondrial disorder to be screened at the MitoRoute for different reasons (e.g., new diagnosis, two-yearly screening, or a deterioration in the clinical condition). Although not all parents and/or children with mitochondrial disorder agree to participate in the MitoRoute, it is not sure whether and how this will lead to selection bias. Despite this, we think that the findings may provide an overview of activity categories for possibly all children with mitochondrial disorders. Future research should include a representative sample, on all characteristics, of children with mitochondrial disorders.

The clinical implications of the large variation of reported activities found in this study could be that health care professionals need to consider all nine activity categories in their assessment and therapy. In addition, clinicians should consult children on the meaning they attach to an activity to decide on activity category to which it belongs. Furthermore, assessments and interventions should be tailored enough to encompass the diversity in activities performed as seen in the global functional capacity profiles of the children and the individual preferences in performance of activities.

## 5. Conclusions

This exploratory pilot retrospective chart review gives insight into the everyday activities performed by children with mitochondrial disorders as reported by professionals. Nine activity categories were identified that represent regular childhood activities. We found that, although the children varied in capacities and limitations, all activity categories were present in each global-functioning profile. There are indications that children perform activities differently in terms of the level of independence, assistive device usage, and energy costs. This study provides a foundation for a qualitative study on the activities of children with a mitochondrial disorder and whether allied health care professionals support their practice. Furthermore, it supports the need for personalized and strength-based care, which puts emphasis on involving the children's perspective in care.

## Figures and Tables

**Table 1:** Clinical and genetic characteristics of the children.

Gender (A)	Age (years)	Level of cognitive development (B)	Level of motor development (C)	Score on Hoffer scale (D)	Level of speech ability (E)	Level of functional abilities of daily activities (F)	Fatigue score (G)	Gene (H)	Mutation
Profile: global low functioning (*N* = 5)									
F	5	1	2	1	4	2	71	*OPA1*	c.910C>T
M	6	1	3	1	1	1	40	*FBXL*	c.1361A>C
M	12	1	2	2	3	2	49	*SDHA*	c.64-2A>G
M	18	1	2	1	3	3	?	*RARS2*	c.442A>G; c.1519G>A
M	18	1	2	3	3	2	49	*ATP6*	m.09176T>C

Profile: low cognitive functioning and moderate to normal ambulation (*N* = 5)									
M	13	2	4	4	6	4	58	*MT-TL1*	m.3243A>G
F	13	1	2	5	5	3	?	*RARS2*	c.442A>G; c.1519G>A
M	14	1	3	5	3	2	49	*MTFMT*	c.626C>T; c.766C>T
M	16	1	3	4	2	3	32	*ATP6*	m.8993T>G
F	17	1	3	5	6	4	54	*MT-TL1*	m.3243A>G

Profile: global moderate functioning (*N* = 7)									
M	4	3	3	4	5	3	65	*TAZ*	c.788_794del
M	4	3	4	4	3	3	72	*ATP6*	m.8993T>G
F	5	3	3	4	6	3	72	*NDUFS7*	c.364G>A
F	5	3	4	4	6	3	39	*MT-TL1*	m.3243A>G
M	7	3	2	4	6	3	66	*ATP6*	m.09185T>C
M	7	3	4	4	6	3	38	*mt-tRNASer*	m.7507A>G
M	13	3	3	4	5	4	58	*MT-TL1*	m.3243A>G

Total									
F: *N* = 5 M: *N* = 12	Range: 4–18	1: *N* = 9 2: *N* = 1 3: *N* = 7 4: *N* = 0	1: *N* = 0 2: *N* = 6 3: *N* = 7 4: *N* = 4 5: *N* = 0	1: *N* = 3 2: *N* = 1 3: *N* = 1 4: *N* = 9 5: *N* = 3	1: *N* = 1 2: *N* = 1 3: *N* = 5 4: *N* = 1 5: *N* = 3 6: *N* = 6	1: *N* = 1 2: *N* = 2 3: *N* = 11 4: *N* = 3	Range: 32–72 2 unknown		

A: M = male, F = female; B: 1 = extremely low, 2 = low, 3 = average, 4 = good; C: 1 = spasticity, 2 = ataxia, 3 = extremely delayed, 4 = delayed, 5 = normal development; D: 1 = nonambulation, 2 = nonfunctional ambulation, 3 = household ambulation, 4 = community ambulation, 5 = normal ambulation; E: 1 = no oral communication possible, 2 = communication with a known person, 3 = frequent repetitions, 4 = incidental repetitions, 5 = effective despite small problems, 6 = effective communication; F: 1 = not self-independent, 2 = minimal self-independent, 3 = sufficient self-independent, 4 = fully self-independent; G: the higher the score, the better the (by fatigue impacted) health-related quality of life, unknown = missing data; H: OPA1 = optic atrophy 1; FBXL = F-box and leucine-rich repeat proteins; SDHA = succinate dehydrogenase complex, subunit A; RARS2 = arginyl-tRNA synthetase 2; ATP6 = ATP synthase 6; MT-TL1 = mitochondrial tRNA leucine; MTFMT = mitochondrial methionyl-tRNA formyltransferase; TAZ = taffazin; NDUFS7 = NADH-ubiquinone oxidoreductase Fe-S protein 7; mt-tRNASer = mitochondrial tRNA serine.
